# Position statement of the Mexican Association of Pediatrics on the immunoprevention of respiratory syncytial virus infection during pregnancy and infancy

**DOI:** 10.1007/s12519-025-00997-1

**Published:** 2025-11-24

**Authors:** Claudia Montesinos Ramírez, Patricia Saltigeral Simental, Federico Javier Ortiz Ibarra, Martha Josefina Avilés Robles, César Adrián Martínez Longoria, Rodolfo Norberto Jiménez-Juárez, José Alberto Castillo, Zigor Campos Goenaga, Carlos López Candiani, Alfonso Copto García, Elsa Diaz López, Vianey Escobar Rojas, María del Carmen Gorbea Robles, Georgina Hernando Becerra, Patricia Laurean Ibarra, Antonio Luévanos Velázquez, Brandon Ortiz-Casas, Francisco Javier Otero Mendoza, Mitchel Martín Padilla Rojas, José Luis Pinacho Velázquez, Lucila del Carmen Sánchez Ortiz, Silvia F. Torres Lira, Víctor Saúl Vital Reyes

**Affiliations:** 1 Mexican Association of Pediatrics, Mexico City, Mexico; 2 Department of Infectious Disease, National Pediatrics Institute, Mexico City, Mexico; 3 Hospital Ángeles del Pedregal, Camino Sta. Teresa 1055-S, Heroes de Padierna, La Magdalena Contreras, 10700 Mexico City, Mexico; 4 Mexican Association of Pediatric Infectious Diseases, Mexico City, Mexico; 5 Service of Infectious Diseases, Hospital Infantil de México Federico Gómez, Mexico City, Mexico; 6 School of Medicine and Health Sciences, Tecnologico de Monterrey, Monterrey, Mexico; 7 Department of Infectious Diseases, Hospital Infantil de México Federico Gómez, Mexico City, Mexico; 8 Faculty of Medicine, National Autonomous University of Mexico, Mexico City, Mexico; 9 Hospital Ángeles Acoxpa, Mexico City, Mexico; 10 High Specialty Medical Unit, IMSS Gynecology and Obstetrics Hospital No. 4 “Luis Castelazo Ayala”, Mexico City, Mexico; 11 Mexican College of Obstetrics and Gynecology Specialists, Mexico City, Mexico; 12 Medical Society of Hospital Ángeles México, Mexico City, Mexico; 13 ISSSTE Pediatric Society, Mexico City, Mexico; 14 Infectious Disease Hospital, IMSS Centro Médico La Raza, Mexico City, Mexico; 15 Mexican Academy of Pediatrics, Mexico City, Mexico; 16 Society of Pediatricians and Neonatologists, “Professor Dr. José Iglesias Leboreiro”, Mexico City, Mexico; 17 Mexican Society of Pediatrics, Mexico City, Mexico; 18 Guadalajara Civil Hospital, Guadalajara, Mexico; 19 Mexican Council of Certification in Pediatrics, Mexico City, Mexico; 20 Department of Science, Tecnológico de Monterrey, Campus Ciudad de México, Mexico City, Mexico; 21 Hospital Ángeles Lindavista, Mexico City, Mexico; 22 National Academy of Medicine of Mexico, Mexico City, Mexico

**Keywords:** Immunoprophylaxis, Monoclonal antibodies, Position statement, Respiratory syncytial virus, Vaccination and pregnancy

## Abstract

**Background:**

Respiratory syncytial virus infection is one of the leading causes of morbidity and mortality in children under two years of age. Thus, the objective of this consensus document is to analyze and discuss current scientific evidence and generate recommendations that reflect the position of a prominent national pediatric professional association regarding the benefits and impact of immunoprophylaxis in pregnant women and infants on the burden of respiratory syncytial virus-related respiratory disease in Mexico and Latin America.

**Data sources:**

Following an academic consensus model, the available scientific literature on current or controversial topics was compiled and critically analyzed. The process adhered to the Guidelines for the Development of Consensus Documents and incorporated recommendations and critical appraisal criteria from the European Appraisal of Guidelines for Research and Evaluation initiative. Multidisciplinary and representative teams were formed from several national professional associations. The studies and manuscripts included in this review were selected via keywords such as respiratory syncytial virus, vaccine, monoclonal antibodies, maternal vaccine, safety and efficacy in vaccine, and vaccine compliance across databases, with priority given to articles published between January 2019 and May 2025. The process included one in-person meeting and one virtual meeting.

**Results:**

A total of nine questions, which were considered unresolved in previous respiratory syncytial virus-related consensus documents, were formulated, covering the time, dose, cost-effectiveness and other perspectives. Each one was addressed through an updated literature review and critical appraisal. The findings and resulting recommendations were presented along with their corresponding level of evidence according to Grading of Recommendations Assessment, Development, and Evaluation.

**Conclusions:**

Both maternal respiratory syncytial virus vaccination (RSVpreF) and passive immunization with nirsevimab demonstrate robust efficacy and favorable safety, protecting newborns and infants from severe respiratory syncytial virus infections. The document provides actionable, evidence-based recommendations tailored for the Latin American healthcare context, aiming to reduce severe respiratory syncytial virus disease incidence and improve national infant health outcomes.

**Graphical abstract:**

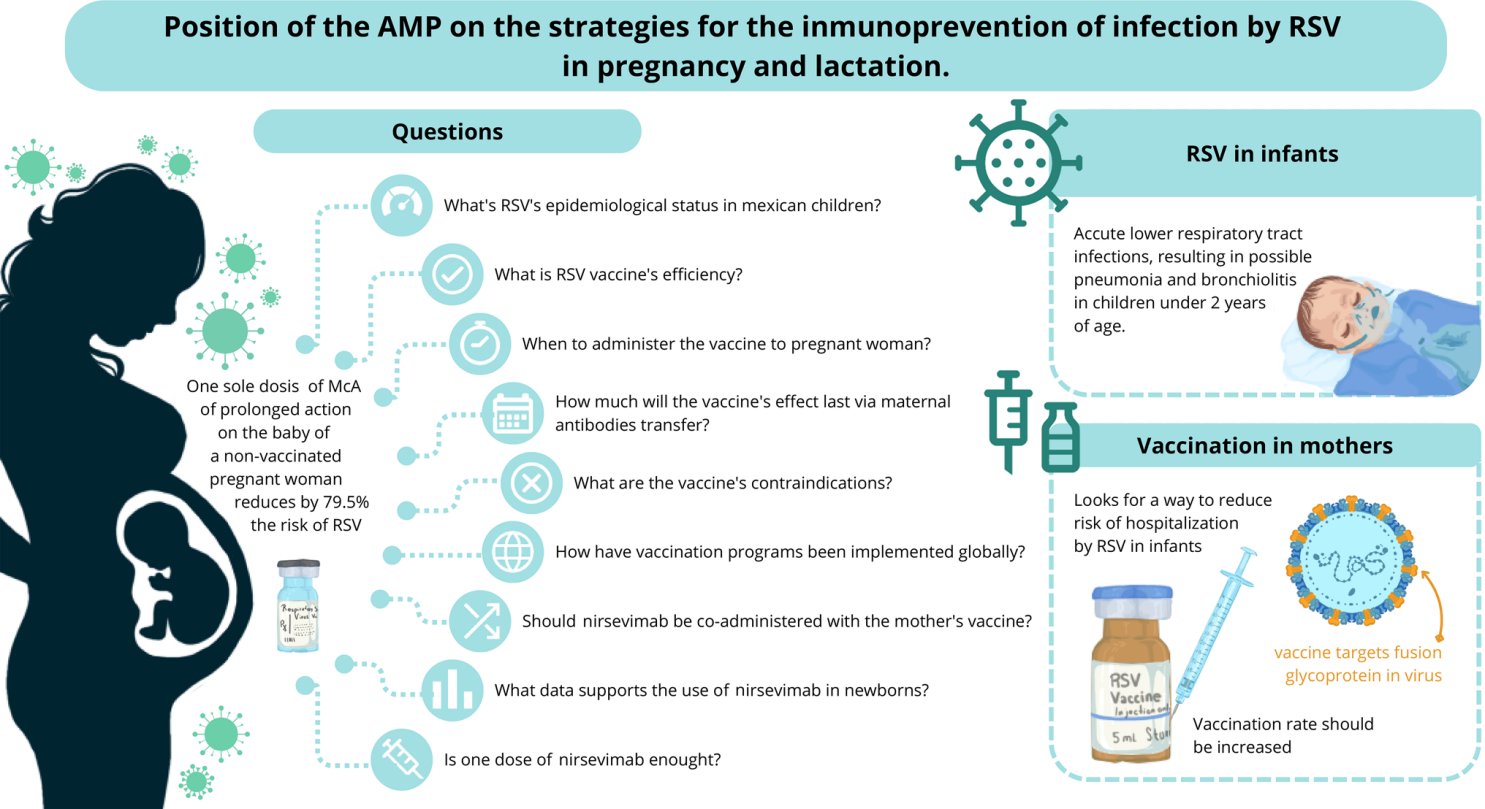

## Introduction

The World Health Organization (WHO) considers respiratory syncytial virus (RSV) one of the most common causes of acute lower respiratory tract infections (LRTIs) in children worldwide. It also causes a significant burden of severe respiratory illness in older adults. RSV is estimated to be responsible for more than 3.6 million hospitalizations and about 100,000 deaths in children under five years of age each year, with 97% of these deaths occurring in low-income and middle-income countries, where access to medical care is markedly more limited [1–4]. RSV transmission peaks mainly during the fall and winter months, although the seasonality varies depending on the hemisphere. In the Northern Hemisphere, most cases occur from October to March, whereas in the Southern Hemisphere, the highest incidence occurs from April to September. This seasonal pattern has influenced the regionalization of immunopreventive strategies across different global populations.

In 2024, a new phylogenetic classification was published, designating RSV as *Orthopneumovirus hominis* within Pneumoviridae, comprising two antigenic subgroups: subgroup A (HRSV-A) and subgroup B (HRSV-B) [5].

Globally, this virus causes over 30 million respiratory infections annually in children under five years of age, accounting for 70% of bronchiolitis cases and 25% of pneumonia cases in infants under one year of age. It typically leads to mild to moderate respiratory illness, but in neonates and infants, it can result in severe pneumonia and bronchiolitis, leading to high hospitalization rates. These factors, in turn, generate significant costs and long-term sequelae (such as residual bronchial hyperreactivity), negatively impacting patients’ quality of life [6]. The considerable burden RSV places on global child health has driven the development of strategic programs to reduce its impact. The recent availability of new preventive immunogens has broadened the range of options for implementing such programs.

In recent years, two new immunization strategies have been authorized globally by leading health regulatory agencies to prevent severe RSV disease in infants: (1) maternal immunization, implemented in some countries from 24 weeks of gestation, uses a vaccine, whereas other countries have implemented vaccination protocols starting at later gestational weeks, such as 28 or 32 weeks; (2) administration of long-acting monoclonal antibodies was given to both healthy newborns and those with comorbidities.

Vaccination during pregnancy has become an essential component of prenatal care, offering critical protection to both pregnant women and their fetuses against potentially life-threatening diseases. This strategy focuses on two main objectives: maternal protection against severe complications [e.g., influenza, coronavirus disease 2019 (COVID-19), and rabies after exposure] or death [7–9] and the dual benefit of active maternal immunization and passive protection of the newborn against potentially fatal infections (e.g., tetanus, pertussis, and RSV). The latter is a policy known as the “cocoon strategy” [7, 8].

While international strategies and recommendations provide a critical framework, their implementation requires careful consideration of the local epidemiological context and the specific challenges of the national healthcare system. Acknowledging this need, the Mexican Association of Pediatrics (AMP), in collaboration with other prominent medical local associations, has reaffirmed the importance of promoting all recommended and authorized vaccines during pregnancy. Unfortunately, vaccination coverage among pregnant women in Mexico remains low, ranging from 55% to 70%, causing concern in several states about the urgent need to improve these rates [10]. This situation calls for a committed response to analyze and disseminate scientifically grounded recommendations, presenting our position as a specialized academic association regarding the benefits of immunizing pregnant women and expanding protective coverage for them and their children. Improving access to these new interventions for maternal and infant well-being and supporting the adoption of globally recognized public health strategies and policies are also essential.

This academic statement was, therefore, developed as an initiative by the AMP, reaffirming its commitment to generating and disseminating up-to-date scientific knowledge among the Mexican and Latin American pediatric communities. These findings respond both to the increasing identification of RSV as a leading cause of acute respiratory infections in our country’s pediatric population and to the emergence of new preventive tools to reduce the impact of this infection during the perinatal and infant stages. Specifically, this consensus aims to present the potential impact of maternal active/passive immunoprophylaxis against RSV infection during the first six months of an infant’s life. It also seeks to showcase the real-world utility of new neonatal prevention strategies (particularly the use of long-acting monoclonal antibodies) in reducing the burden of RSV-related respiratory disease in Mexico and Latin America. With these aims, this statement aims to provide evidence-based answers and recommendations regarding unresolved questions or controversies that have not yet been addressed in previous consensuses [11–13].

## Methods

### Expert panel formation

A multidisciplinary expert panel comprising 23 specialists was rigorously selected by the Mexican Association of Pediatrics (AMP). The panel members were chosen on the basis of their recognized clinical and/or research expertise related to respiratory syncytial virus (RSV) prevention and care, as well as their expertise in research methodology. The selected specialists represent a wide range of academic and clinical backgrounds in pediatrics, neonatology, pediatric infectious diseases, and obstetrics–gynecology. The composition was meticulously designed to ensure a balanced and comprehensive representation of the national healthcare and academic landscape. This included specialists from both the public sector and leading private institutions, as well as members from prominent public and private medical colleges of the country. Furthermore, the panel also benefited from the participation of members from various medical societies, ensuring a broad and fair representation of perspectives.

### Consensus meeting and scope definition

The expert panel initially met in person to establish the objectives, define the scope of the consensus recommendations, and identify key topics for discussion. During this meeting, the panel was divided into three specialized working groups, each with a set of topics on the basis of their collective expertise, and designated coordinators to facilitate subsequent collaborative work. Each group was collectively responsible for conducting a focused literature search on their assigned topics and drafting their respective portions of the manuscript. The group coordinators served to lead the discussions, synthesize the findings, and oversee the initial manuscript draft.

Criteria were also established for the adoption and adaptation of existing recommendations from other international organizations and medical societies. This approach followed recommendation guidelines such as those established by the Appraisal of Guidelines for Research and Evaluation (AGREE) European initiative for critical reading of scientific articles [14, 15].

### Literature search strategy

A systematic literature search was conducted by the three working groups via a combination of keywords, including “respiratory syncytial virus,” “vaccine,” “maternal immunization,” “epidemiology,” “safety and efficacy vaccine,” “compliance vaccine” and “monoclonal antibodies RSV.” Publications in English and Spanish were reviewed from the PubMed, Embase, Cochrane Library, and Google Scholar databases. Additional scientific databases accessible through Tecnológico de Monterrey were also utilized. Priority was given to the literature published between January 2020 and June 2025; however, key older references were included for their foundational or historical relevance. A total of 74 documents were thoroughly analyzed, including original research articles; systematic reviews; meta-analyses; consensus statements; expert opinion documents; and relevant institutional, governmental, or media publications. This analysis specifically incorporated three recent consensus statements from Latin American expert groups [11–13]. Further citation searching (snowballing) was performed on identified references to capture relevant long-term follow-up studies.

### Evidence synthesis and consensus process

On the basis of the initial literature review and expert input, nine key clinical questions or unresolved issues were identified, drawing from prior consensuses and recent evidence. These questions were structured via a systematic and critical methodology [16]. The evidence-based answers to these questions were then drafted through collaborative work within the three dedicated discussion groups. The first draft of the manuscript was prepared by the consensus coordinators and subsequently remotely reviewed by the full working group. The project coordinators provided final approval of the manuscript after a comprehensive review.

### Grading of recommendations

All the recommendations presented in this consensus statement are accompanied by their respective levels of evidence and strength of recommendation, as determined by a modified Grading of Recommendations Assessment, Development and Evaluation (GRADE) framework [17] (Table 1).

**Table 1:** Modified Grading of Recommendations Assessment, Development and Evaluation (GRADE) framework for recommendations

Quality of evidence	Interpretation
1A	The benefits clearly outweigh the risks and burdens, or vice versa. CTs without major limitations or overwhelming evidence from observational studies. Strong recommendation: can be applied to most patients in most circumstances, without reservation
1B	The benefits clearly outweigh the risks and burdens, or vice versa. CTs with important limitations (inconsistent results, methodological flaws, indirect or imprecise evidence) or exceptionally strong evidence from observational studies. Strong recommendation: can be applied to most patients in most circumstances without reservation
1C	The benefits clearly outweigh the risks and burdens, or vice versa. Observational studies or case series. Strong recommendation: but may change when more high-quality evidence becomes available
2A	Benefits closely balanced against risks and burden. CTs without major limitations or overwhelming evidence from observational studies. Weak recommendation: the best action may vary, depending on the patients’ circumstances or the values of society
2B	Benefits closely balanced against risks and burden. CTs with important limitations (inconsistent results, methodological flaws, indirect or imprecise) or exceptionally strong evidence from observational studies. Weak recommendation: the best action may vary, depending on the patients’ circumstances or the values of society
2C	Uncertainty in estimates of benefits, risks and burdens. Benefits, risks, and burden may be closely balanced. Observational studies or case series. Very weak recommendations: other alternatives may be equally reasonable

*CT* clinical trial

## Epidemiology and burden of respiratory syncytial virus infection

### Question 1: What is the epidemiological status and burden of respiratory syncytial virus infection in Mexican infants?

Globally, RSV causes about 33 million cases of LRTIs, of which 10% (3.2 million) require hospitalization, resulting in more than 100,000 deaths in children under five years of age. This virus affects primarily infants under six months of age, who account for nearly half of all RSV-related deaths. More than 97% of RSV deaths occur in low-income and middle-income countries [1, 2].

In Mexico, RSV data are derived primarily from official reports by the General Directorate of Epidemiology (Dirección General de Epidemiología). In addition to the surveillance of influenza and SARS-CoV-2, other respiratory viruses are grouped under the category of “other respiratory viruses” (ORVs), which includes RSV. According to these reports, a total of 7119 positive cases of ORV were confirmed during the 2024–2025 season, with RSV accounting for 48.0% of the cases. The remaining cases were caused by enterovirus, rhinovirus, metapneumovirus, parainfluenza, adenovirus, and endemic coronaviruses. RSV displays seasonal peaks in autumn and winter, usually starting a few weeks earlier than influenza does and continuing into late April 2025 [18]. In tropical areas, it circulates at lower levels year-round, which is likely linked to periods of higher humidity. The main age group affected included children under six years of age with bronchiolitis and pneumonia. It is estimated that 50% of children are infected with RSV in their first year of life, and most have encountered the virus by age two [18].

In 2017, Wong et al. published a multicenter study assessing the frequency of respiratory viruses in children under five years of age who were diagnosed with pneumonia across 11 hospitals in Mexico. In this study, 1404 patients met the inclusion criteria. Virus detection via polymerase chain reaction (PCR) identified respiratory viruses in 81% of the samples, with RSV being the most common; RSV was detected in 23.7% of all community-acquired viral pneumonias in children under five years of age [19]. Risk factors for severe pneumonia include the use of biomass for cooking, lack of breastfeeding, daycare attendance, and viral coinfections [19]. With the onset of the COVID-19 pandemic in 2020, the number of RSV cases significantly declined. However, after public health restrictions were lifted, an extraordinary surge occurred in the summer of 2021. In the 2022–2023 season, a higher incidence was observed in 12–24-month-old children, likely due to “immune debt” [20]. A 2025 interdisciplinary consensus led by Wong reported that, in Mexico, comorbidities increase the risk of RSV infection [odds ratio (OR) 2.56]. However, 70% of hospitalized children are otherwise healthy, indicating that severe disease can occur even in infants without underlying conditions [13].

RSV has been identified as the causative agent in 30%–60% of LRTIs requiring hospitalization (four times more frequently than influenza), particularly in infants aged 1–6 months. An observational study from a Mexican medical center in 2022 revealed RSV in 44% of hospitalized pediatric patients with respiratory symptoms, with a mean age of 17.5 months. In 2023, Mexico’s National Epidemiological Surveillance System (SINAVE) reported an RSV prevalence of 64% in this age group. These findings confirm that RSV is a significant public health challenge.

In terms of global mortality, LRTIs are the leading cause of death in children under one year of age, and one in every 50 deaths (from any cause) in children under five years of age is attributable to RSV. Mortality is higher in low-income countries, including Mexico. Among hospitalized patients, mortality ranges between 1% and 5%, particularly in those with heart disease, prematurity, or lung disorders. A systematic review by Shi et al., which included 250 studies, estimated the global RSV incidence, hospitalization, and mortality. Among these studies, 90 included incidence data, 103 reported mortality rates, and 218 reported the proportion of hospitalized children with RSV-related LRTIs. For 2015, the study estimated 33.1 million acute LRTI cases due to RSV [uncertainty range (UR): 2.7–3.8], 3.2 million hospitalizations (UR: 2.7–3.8) and 59,600 deaths (UR: 48,000–745,000) in children under five years of age. Among infants under six months of age, there were 1.4 million hospitalizations (UR: 1.2–1.7) and 27,300 in-hospital deaths (UR: 20,700–36,200) [21]. Other studies in Mexico, such as one published by the Mexican Emerging Infectious Disease Clinical Research Network (La Red), assessed hospitalization risk by virus type in children with influenza-like illness between 2010 and 2014. Compared with other viruses, RSV is the pathogen most commonly associated with increased hospitalization risk in 1486 children under five years of age [22]. Vizcarra et al., in San Luis Potosí state, analyzed pediatric intensive care unit (PICU) admission and mortality rates from 2003 to 2014 in children hospitalized for RSV-related acute LRTI. The PICU admission rate was 5.2%, and the mortality rate was 1.04%. They analyzed 1153 patients with RSV, with available data on underlying disease and hospital outcomes. Sixty children were admitted to the PICU, and 12 died. Relevant comorbidities were found in 320 patients. Infants with a history of prematurity and respiratory disorders (excluding asthma) had higher PICU admission rates. Mortality was highest in infants with respiratory, cardiac, or neurological disorders. PICU admission and mortality rates are also highest in infants under six months of age [23].

## Maternal immunization strategies for respiratory syncytial virus prevention

### Question 2: What is the demonstrated efficacy and safety of the respiratory syncytial virus vaccine in preventing serious respiratory syncytial virus infections in newborns and infants?

Currently, the most robust evidence of maternal RSV vaccine efficacy in preventing severe infections in newborns and infants comes from the bivalent RSVpreF vaccine, which targets the prefusion F protein of the virus (Table 2).

**Table 2:** Inter-study efficacy/effectiveness comparison over time of the RSVpreF vaccine

Trial	Type	Vaccine efficacy against severe LRTI 90/180 days	Vaccine efficacy against hospitalization	Effectiveness against hospitalization
Kampmann and Madhi, 2023	Controlled clinical trial	81.8% (99.5% CI 40.6%–96.3%) for 90 d 69.4% (97.58% CI 44.3%–84.1%) for 180 d	–	–
Razzini et al., 2025	Retrospective cohort	–	Largest decrease in RSV hospitalization by 33.57% (95% CI 29.48%–37.17%)	–
MATISSE Study, 2025	Phase 3 controlled clinical trial	81.8% (99.5% CI 40.6%–96.3%) for 90 d 70.0% (95% CI 50.6%–82.5%) for 180 d	–	–
BERNI Study, 2025	Real world evidence. Multicenter, retrospective, case–control	–	–	78.6% (95% CI 62.1%–87.9%) for 90 d 71.3% (95% CI 53.3%–82.3%) for 180 d

*LRTI* lower respiratory tract infection, *CI* confidence interval, – not available

The initial study by Kampmann and Madhi demonstrated a vaccine efficacy of 81.8% [99.5% confidence interval (CI) 40.6%–96.3%] in preventing severe LRTI during the first 90 days of life and 69.4% efficacy (97.58% CI 44.3%–84.1%) at 180 day post-birth. For RSV-related medically treated LRTIs, the efficacy at 90 days was 57.1% (99.5% CI 14.7%–79.8%) [24].

In 2025, Razzini et al. reported a retrospective cohort study in Argentina evaluating the benefit of maternal RSV vaccination. They reported a 33.57% reduction in hospitalizations (95% CI 29.48%–37.17%), with a number needed to immunize (NNI) of 83.87 (95% CI 65.91–185.38) to prevent one hospitalization [25].

The maternal immunization study for safety and efficacy (MATISSE) phase 3 trial, published in 2025, also using the bivalent RSVpreF vaccine, reported 81.8% efficacy (99.5% CI 40.6%–96.3%) against severe RSV–LRTIs at 90 days of life and 70.0% efficacy (95% CI 50.6%–82.5%) at 180 days. Efficacy against RSV–LRTIs requiring medical care was 57.1% (99.5% CI 14.7%–79.8%) at 90 days [26].

This efficacy has been powerfully validated in real-world settings. In May 2025, BERNI, a real-world effectiveness study conducted during the 2024 RSV season in Argentina, was published. Argentina became the first Latin American country to implement a national maternal immunization program using RSVpreF as the main strategy to prevent RSV disease in infants. This was a retrospective multicenter case‒control study with RSV-negative patients from 12 hospitals. Among 633 infants hospitalized for LRTI (April 1–September 30, 2024), 505 were eligible (286 cases and 219 controls). Eighteen percent of the cases and 50% of the controls were born to mothers vaccinated with RSVpreF during pregnancy. Vaccine effectiveness against RSV-associated LRTI leading to hospitalization was 78.6% (95% CI 62.1%–87.9%) from birth to three months; 71.3% (95% CI 53.3%–82.3%) from birth to six months; and against severe RSV–LRTI requiring hospitalization, the effectiveness was 76.9% (95% CI 45.0%–90.3%) from birth to six months. Three RSV-related in-hospital deaths occurred, all in infants whose mothers were not vaccinated during pregnancy. These real-world data from Argentina’s 2024 RSV season demonstrate that RSVpreF is highly effective in preventing both RSV-related LRTI and severe cases leading to hospitalization, particularly during the first three months of life, with protection maintained for up to six months [27].

In terms of safety, a complete benefit‒risk profile requires a comprehensive review of all available clinical data. An analysis of the MATISSE trial revealed that common side effects were generally mild to moderate, which is consistent with other vaccines. Nonetheless, the main highlight of security concerns lies in the risk of preterm birth (PTB). The overall trial revealed a statistically significant increase in PTB rates among vaccinated individuals (5.7%) compared with the placebo group (4.7%). However, a crucial subgroup analysis revealed a geographical disparity, with a statistically significant imbalance in non-high-income countries, where the rates were 7.0% in the vaccinated group versus 4.0% in the placebo group. Importantly, this imbalance was not observed in high-income countries [28]. These discrepancies can also be attributed, at least partially, to underlying health disparities and confounding factors and not necessarily to a direct, causal effect of the vaccine. Therefore, more clinical evidence is needed to confirm or reject any conclusions.

On the other hand, this observed geographical discrepancy in the PTB safety signal could have led to divergent, yet rational, regulatory responses from global health authorities. The US Food and Drug Administration (FDA) and its Advisory Committee on Immunization Practices (ACIP) opted for a more risk-averse approach, recommending a narrower vaccination window of 32–36 weeks to mitigate the potential, albeit still unproven, risk of preterm births [29]. In contrast, the European Medicines Agency (EMA) and the WHO adopted broader perspectives (24–36 weeks and 28+ weeks, respectively), choosing to weight the substantial and proven benefits of the vaccine against an unproven risk signal within high-burden global contexts [30, 31]. This divergence in recommendations highlights how evidence may be interpreted differently on the basis of both the epidemiology of their target population and their regional-specific risk–benefit philosophies.

Furthermore, postlicensure surveillance systems play a crucial role in detecting rare adverse events. While the BERNI study’s primary objective was to measure effectiveness rather than safety, its findings are crucial for overall benefit–risk assessment. Global postmarketing surveillance has not corroborated the preterm birth signal, reinforcing the conclusion that the benefits of maternal RSV immunization overwhelmingly outweigh the potential, unproven risks.

### Question 3: What is the optimal gestational age for administering the respiratory syncytial virus vaccine in pregnant women?

The optimal gestational age for RSV vaccine administration is a frequent point of discussion, driven by differing recommendations across various gestational weeks, as established by international regulatory bodies in their approvals for the RSVpreF vaccine.

On August 26, 2023, FDA, through the Division of Vaccines and Related Products Applications Office of Vaccines Research and Review, approved the use of the RSVpreF vaccine for pregnant women between 32 and 36 weeks of gestation to prevent RSV-related LRTIs in infants under six months of age [32]. In July 2024, the EMA granted marketing authorization in the European Union (EU) for this vaccine as an immunization to protect against disease caused by respiratory syncytial virus. This is the first RSV vaccine indicated for the passive immunization of infants from birth to six months of age following maternal vaccination during pregnancy, between 24 and 36 weeks of gestation. This vaccine was also approved for active immunization of adults aged 60 years and older [30].

In Mexico, on December 16, 2024, the Federal Commission for Protection against Health Risks (Comisión Federal para la Protección contra Riesgos Sanitarios or COFEPRIS) announced that it had granted two registrations for the recombinant bivalent vaccine against RSV. It was authorized for the prevention of respiratory diseases caused by RSV in infants from zero to six months of age through maternal vaccination during pregnancy between 24 and 36 weeks of gestation. This vaccine was also approved for the prevention of acute respiratory disease and lower respiratory tract disease caused by RSV in people aged 60 years and older [33]. Although these approvals come with differing recommendations, they are based on the available safety and efficacy data presented at the time of application, as supported by clinical trial findings. Nevertheless, this consensus holds that such guidance should be complemented with a stronger immunological basis, supported by current scientific knowledge.

Transplacental transfer of immunoglobulin G (IgG) during pregnancy provides passive immunity to the newborn and is crucial for protection against infections in the early stages of life. The placenta is an immunologically active organ that interacts with pathogens and modulates the maternal immune response [34]. The transfer of maternal antibodies across the placenta depends on the neonatal Fc receptor (FcRn), which is expressed in the syncytiotrophoblast. IgG antibodies are selectively transported from maternal blood to the fetus via this receptor [8, 35]. FcRn levels are low during the first 20–22 weeks of pregnancy but increase significantly by the third trimester, which begins at week 28 of gestation. Therefore, the role of FcRn in transferring maternal IgG antibodies across the placenta is essential for fetal protection [8, 35], explaining why IgG transfer before this gestational age is minimal.

Vaccination during pregnancy increases maternal vaccine-specific antibody concentrations; thus, the amount transferred across the placenta to the infant should follow these principles [36, 37]. Active transplacental IgG transfer begins at 13 weeks of gestation and reaches optimal levels after week 32. This mechanism serves as a specific adaptation that compensates for newborns’ and infants’ limited antibody production in the early postpartum months, providing temporary passive immunity [38, 39].

During the first and second trimesters, the concentration of fetal antibodies after maternal vaccination is significantly lower than the maternal level. This increases to 50% of the maternal blood antibody concentration from week 28 to 32, with levels in fetal and maternal blood equalizing by week 36. In addition, since maternal IgG-specific levels peak around four weeks after immunization and FcRn levels in syncytiotrophoblasts reach ideal concentrations starting in the third trimester, gestational age at vaccination is a critical factor for achieving neonatal passive immunity [40]. Therefore, on a solid immunological basis, this consensus holds that the ideal time for maternal vaccination during pregnancy is at 28 weeks of gestation to achieve maximum maternal antibody levels and maximum transfer delivery [41].

### Question 4: What is the estimated duration of protection against respiratory syncytial virus conferred by transplacental transfer of maternal antibodies?

To answer this question, a phase 2 randomized, double-blind, placebo-controlled clinical trial (NCT04126213) was conducted. In this trial, the RSVPreF3 vaccine (60 or 120 µg) was administered during the second or third trimester of pregnancy to 213 healthy women aged 18–40 years. Serum antibody titers were measured before vaccination, and both mothers and their infants were followed for six month post-partum. Immunogenicity was assessed one month after vaccine administration and on days 43 and 181 postpartum in both dose groups (60 µg and 120 µg) and in the placebo group. The RSVPreF3 vaccine was well-tolerated. In the 60 and 120 µg RSVPreF3 groups, neutralizing antibody titers in mothers increased 12.7-fold and 14.9-fold against HRSV-A and 10.6-fold and 13.2-fold against HRSV-B, respectively, one month after vaccination. These levels remained elevated by 8.9-fold (HRSV-A) and 10.0-fold (HRSV-B) on day 43 postpartum compared with prevaccination levels. Neutralizing antibody titers were consistently higher than those observed in the placebo group. The placental transfer rates of anti-RSVPreF3 antibodies at birth were 1.62 and 1.90 for infants born to mothers who received 60 and 120 µg, respectively, and these levels decreased by day 181 postpartum. The authors concluded that maternal vaccination with RSVpreF had an acceptable safety profile and induced robust RSV-specific immune responses, with successful antibody transfer to newborns, which was maintained for up to 81 days of life [42].

A meta-analysis evaluating the safety, immunogenicity, and efficacy of the RSVpreF vaccine in infants and their mothers reviewed six randomized clinical trials. Maternal serum neutralizing antibody geometric mean titers (GMTs) against RSV were assessed. Before vaccination, the pooled standardized mean differences (SMDs) of the GMTs for RSV subtype A (HRSV-A) and subtype B (HRSV-B) were − 0.30 (95% CI − 0.78–0.19) and 0.00 (95% CI − 0.38–0.38), respectively. After vaccination, the pooled SMDs for HRSV-B were 3.4 (95% CI 1.35–5.45) and 1.67 (95% CI 0.26–3.08), respectively, at the time of delivery. The meta-analysis also revealed that the SMDs of GMTs for prefusion F protein-specific antibodies before immunization and at delivery were − 0.32 (95% CI − 0.91–0.22) and 7.48 (95% CI 3.13–11.83), respectively, for HRSV-A and HRSV-B.

According to this meta-analysis, the cord blood GMTs of infants born to vaccinated mothers were 2.67 (95% CI 1.12–4.22) and 1.22 (95% CI 0.26–2.18) for HRSV-A and HRSV-B, respectively. The pooled SMD for anti-RSV F protein-specific IgG (F-IgG) antibodies was 1.49 (95% CI 1.42–1.56) compared with neutralizing antibodies in newborns from unvaccinated mothers [43]. Among the studies included, three reported maternal vaccine effectiveness in reducing the incidence of severe lower respiratory tract infections requiring medical attention and RSV-related hospitalizations. Compared with those in the control groups, there was a 53% reduction in the risk of severe lower respiratory tract infection (OR 0.47, 95% CI 0.23–0.98) and a 49% reduction in RSV-related hospitalizations (OR 0.51, 95% CI 0.38–0.67) among infants born to vaccinated mothers within the first six months of life. This consensus suggests that prenatal RSV vaccination not only is safe and immunogenic in pregnant women but also provides effective levels of antibodies in infants and reduces severe RSV-related illness in children under six months of age [43].

### Question 5: What are the specific contraindications for respiratory syncytial virus immunization during pregnancy?

There are few absolute contraindications for maternal RSV immunization. However, individual clinical assessment is essential, considering obstetric factors, relevant medical history, and the immunological profile of the pregnant woman.

1. As with any immunogen, a history of severe allergic reactions (e.g., anaphylaxis) to any component of the vaccine (excipients: trometamol, trometamol hydrochloride, sucrose, mannitol, polysorbate 80, and sodium chloride) is an absolute contraindication for its administration [44]. If such a reaction occurs following vaccination, it must be reported [45].
2. The presence of a fever below 38.5 °C or acute illness is not an absolute contraindication for immunization. In fact, for other vaccines, the Secretariat of Health’s (Secretaria de Salud) vaccination manual categorizes these as “false contraindications.” However, given that this vaccine is not yet part of Mexico’s universal immunization program and that there is limited real-world experience with vaccination during acute illness, this consensus suggests postponing vaccination until the patient recovers if any of these circumstances are present on the scheduled vaccination day.
3. Vaccination is not recommended in women with < 24 weeks of gestation, as its use before this point has not been studied [43, 46]. The evidence regarding safety and efficacy in the first and early second trimesters is still limited, so use should be restricted to later stages of pregnancy.
4. Vaccination is not recommended after 36 weeks and six days of gestation, not because of any associated risk but because there is likely insufficient time. Maternal antibodies require 2–4 weeks after immunization to reach optimal blood concentrations and effectively cross the placenta.

## International implementation of respiratory syncytial virus prevention strategies

### Question 6: How have respiratory syncytial virus vaccination strategies been implemented in maternal immunization programs internationally?

United States: Since 2023, the official recommendation has been to administer a single dose of the RSV vaccine to pregnant women between 32 0/7 and 36 6/7 weeks of gestation. The primary goal of this measure is the prevention of severe RSV-related respiratory illness in infants under six months of age, who represent the group at highest risk for hospitalization. In most parts of the United States, the RSV season extends from September to January. To date, it is not recommended that pregnant women who receive the maternal RSV vaccine receive an additional dose in subsequent pregnancies [7, 47].

Spain: The Vaccine Advisory Committee of the Spanish Association of Pediatrics (Comité Asesor de Vacunas e Inmunizaciones de la Asociación Española de Pediatría or CAV–AEP) recommends the administration of a single dose of the RSV vaccine between 24 and 36 weeks of pregnancy. This committee has emphasized the importance of this preventive intervention, especially during months of high viral circulation. However, as of the time of writing this document, this public health policy has not yet been implemented by the Ministry of Health of Spain [48].

United Kingdom: The inclusion of this vaccine in the public health system reinforces the priority given to neonatal protection through passive immunization strategies. Since September 2024, a single dose of the RSV vaccine has been recommended starting at week 28 of pregnancy, and it may be administered up to the time of delivery. Pregnant women can be vaccinated during each pregnancy. This vaccine is freely available through the National Health Service (NHS) of England [49].

Latin America: This recommendation is aligned with the global strategies of the WHO to reduce childhood morbidity associated with respiratory infections. Since November 2023, the Technical Advisory Group on Immunization of the Pan American Health Organization (PAHO) has recommended the administration of the RSV vaccine to pregnant women between 32 and 36 weeks of gestation to prevent RSV-related illness in infants [50].

Argentina: The maternal RSV vaccination campaign in Argentina began on March 1, 2024, as announced by the Ministry of Health of the Nation. It is part of the National Immunization Schedule, meaning that it is free and mandatory. It is administered to pregnant women between 32 0/7 and 36 6/7 weeks of gestation and is available nationwide during the months of highest RSV circulation, especially between March and July [51].

Uruguay: Since August 2024, the RSVpreF vaccine has been used. The National Vaccine Advisory Commission recommended administering the RSVpreF vaccine to pregnant women over 18 years of age, from 32 to 36 weeks and six days of gestation, with administration on the basis of the timing of viral circulation. The vaccination is universal and free. The 2024 RSV vaccination campaign took place from August 19 to September 30, 2024, and lasted seven weeks. The 2025 campaign began on January 13, 2025, and will run until August 31 [52].

Costa Rica: The National Commission on Vaccination and Epidemiology (Comisión Nacional de Vacunación y Epidemiología) approved the maternal RSV vaccination campaign in November 2024. This strategy targets pregnant women between 32 and 36 weeks of gestation, with the goal of protecting newborns from severe respiratory infections. The vaccine is expected to be available for administration by 2025 [53, 54].

Mexico: The RSV vaccine was authorized by COFEPRIS in December 2024 for use in pregnant women between 24 weeks and 36 + 6 weeks of gestation [33].

## Passive immunization with monoclonal antibodies

### Question 7: Is coadministration of maternal respiratory syncytial virus vaccination and nirsevimab recommended?

The combination of maternal vaccination and the long-acting monoclonal antibody nirsevimab is not necessary in most situations. Infant protection on the basis of maternal vaccination relies on the adequate and sufficient transplacental transfer of antibodies, as has already been demonstrated in countries with greater accumulated experience. In scenarios where such antibody transfer may be diminished, the administration of nirsevimab may be necessary despite the mother having received the RSV vaccine. For example, infants born within 14 days of vaccine administration may present with placental abnormalities or maternal human immunodeficiency virus (HIV) infection. However, these situations are uncommon and should be evaluated individually by healthcare professionals providing care [55, 56]. The decision to administer both interventions should be based on clearly defined clinical criteria and exceptional circumstances that compromise transplacental antibody transfer.

The use of nirsevimab may be considered in infants with certain vulnerabilities, who, in addition to maternal immunization, could benefit from additional protection via monoclonal antibodies [57].

### Question 8: What efficacy and safety data support the use of nirsevimab in healthy term and preterm newborns?

Nirsevimab is a long-acting monoclonal antibody that blocks the F1 and F2 sites of the fusion (F) protein of RSV. The current evidence supporting its utility began to be established on the basis of the following studies.

Hammitt et al. conducted a randomized, double-blind clinical trial in late preterm infants (35–36 weeks of gestational age) and full-term neonates without comorbidities, with the aim of evaluating its efficacy in preventing RSV-related LRTIs requiring medical attention. The secondary outcomes included safety and pharmacokinetics. A total of 1027 infants were randomized at a 2:1 ratio to receive nirsevimab or placebo before the RSV season began. The results revealed 77.2% efficacy at 150 days (95% CI 59.8%–86.8%) and an NNI of 12 to prevent one RSV–LRTI. The efficacy of preventing hospitalization due to RSV–LRTI was 59% (95% CI 2.1%–82.9%), with an NNI of 53 [58].

The group of Simões and collaborators combined data from a phase 2b study to evaluate the relative risk reduction (RRR) for hospitalization due to RSV–LRTI. They also assessed outpatient visits for LRTI and antibiotic use. Nirsevimab led to an RRR of 79.5% (95% CI 65.9%–87.7%) for RSV-related LRTI requiring medical care, an RRR of 77.3% (95% CI 50.3%–89.7%) for RSV–LRTI hospitalizations, and an RRR of 86.0% (95% CI 62.5%–94.8%) for severe RSV–LRTI hospitalizations. Moreover, it caused an RRR of 23.6% (95% CI 3.8%–39.3%) [59].

Drysdale and collaborators conducted a pragmatic, open-label randomized clinical trial to assess the efficacy and safety of nirsevimab in preventing hospitalizations due to RSV-related lower respiratory tract infections. A total of 8058 children under one year of age without comorbidities were included. The efficacy was 83% (95% CI 67.8%–92.0%), and the efficacy against very severe disease hospitalizations (defined as hospitalized subjects with oxygen saturation < 90%) was 75.7% (95% CI 32.8%–92.9%). Stratified analysis revealed that immunization before three months of age provided the greatest benefit, likely due to the decrease in hospitalization rates as age increased [60].

With respect to safety, no significant differences in adverse events were observed between the nirsevimab and placebo groups across the trials. In addition, Domachowske evaluated side effects in a randomized clinical trial in a high-risk population (preterm infants with cyanotic congenital heart disease and bronchopulmonary dysplasia) who were eligible for palivizumab. The infants were randomized 1:1 to receive either palivizumab or nirsevimab. Adverse effects were similar in both groups, with 0.4% of the nirsevimab group and 3.6% of the palivizumab group developing anti-drug antibodies [61]. The systematic review conducted by the ACIP in the United States considered the strength of evidence for benefits to be high [29].

The HARMONIE trial, a phase 3b multicenter, open-label, parallel-arm, randomized (1:1) controlled study conducted in France, Germany and the UK, evaluated infants aged 12 months or younger, born at ≥ 29 weeks of gestation, who received a single intramuscular dose of nirsevimab (50 mg for infants < 5 kg or 100 mg for ≥ 5 kg) or no RSV prophylaxis prior to their first RSV season. The primary outcome was the incidence of RSV-associated lower respiratory tract infection hospitalizations within 180 days after administration. Safety was assessed up to 365 days after administration. Between August 8, 2022, and February 28, 2023, a total of 8057 infants were randomized: 4038 to the nirsevimab group and 4019 to standard care. The median age at randomization was 4 months. By day 180, 12 infants (0.3%) in the nirsevimab group and 68 (1.7%) in the standard care group were hospitalized due to RSV–LRTI, resulting in an efficacy of 82.7% (95% CI 67.8%–91.5%; *P* < 0.001). No apparent safety concerns were observed through day 365 [62].

### Question 9: Is the protection conferred by a single dose of nirsevimab sufficient, and what is its impact on the respiratory syncytial virus-related hospitalization burden?

In addition to the previously presented efficacy trials described, another conducted with a single dose of nirsevimab, aiming to evaluate the effectiveness of nirsevimab in reducing RSV-related LRTI hospitalizations during the RSV season. The study assessed three groups: infants born during the RSV season, infants under six months of age at the beginning of the RSV season, and high-risk children under 24 months of age at the start of the season. The study, which achieved 91.7% coverage of the target cohort, revealed an 89.8% reduction in hospitalizations due to RSV compared with the median of previous seasons. An 86.9% reduction in severe RSV hospitalizations requiring oxygen therapy was also observed. This benefit was observed across the entire cohort and in infants born before or during the RSV season, respectively [63]. The NNI to prevent one RSV hospitalization was 25. The safety profile was appropriate, with no significant adverse events reported [63].

In Catalonia, a similar study immunized 26,525 infants under six months of age and reported an 87.5% reduction in RSV-related hospitalizations and a 90.1% reduction in intensive care unit (ICU) admissions. Additional reductions included 48% in primary care visits due to bronchiolitis, 60% in viral pneumonias of any cause, and 55% in emergency room consultations for RSV-related LRTIs [64].

An estimate from Spain’s Severe Acute Respiratory Infections (SARI) network projected that the introduction of nirsevimab prevented between 9364 and 9875 hospitalizations due to RSV during the 2023–2024 season [65]. Similarly, a rapid evaluation by the Centers for Disease Control and Prevention (CDC) revealed a 90% reduction in RSV hospitalizations with the introduction of nirsevimab [66].

## Role of other potential monoclonal antibodies

In June 2025, the US FDA approved a new long-acting monoclonal antibody for the prevention of RSV in infants: clesrovimab. This marked a significant advancement, providing another monoclonal antibody option to protect newborns during their first RSV season. Following the FDA’s approval, the ACIP recommended clesrovimab as an option for the protection of infants younger than eight months of age who are born during or enter their first RSV season and who have not been protected through maternal RSV vaccination. The ACIP’s recommendation places nirsevimab and clesrovimab as two coequal options, with no preference for one over the other. The choice between the two should be guided by factors such as parental preference, product availability, and the specific timing of the RSV season [67, 68].

The introduction of clesrovimab represents a major step forward, offering an alternative to nirsevimab and potentially replacing the need for palivizumab, which would be discontinued at the end of 2025 [69].

## Recommendations

### Optimal gestational age for maternal respiratory syncytial virus vaccination

The goal of this consensus is to provide adequate protection to both preterm and full-term newborns born during the RSV seasonal circulation period in our country. Therefore, we recommend the vaccination of pregnant women against RSV during an immunologically optimal period, starting at 28 weeks of gestation (quality of evidence: 1A).

### Maternal respiratory syncytial virus vaccination beyond 36 weeks + 6 days gestation

Vaccination against RSV is not recommended for pregnant women beyond 36 weeks and 6 days of gestation, as there is unlikely to be sufficient time for antibody development, transplacental transfer, and effective protection of the newborn or infant. Instead, newborns should receive RSV prophylaxis with the long-acting monoclonal antibody nirsevimab just before or at the start of the RSV season (quality of evidence: 1A).

### Respiratory syncytial virus prophylaxis for newborns

Currently, if a pregnant woman has received the vaccine and it has been < 14 days; the infant is born prematurely; or the mother is immunocompromised, and protection is needed for these newborns in the absence of nirsevimab, clesrovimab could be considered if it is already regulatory-adopted and commercially available in the corresponding region (quality of evidence: 1A).

### No repetition of maternal respiratory syncytial virus vaccination in subsequent pregnancies

Currently, if a pregnant woman has already received maternal RSV vaccination in a previous pregnancy, both the US CDC and the American College of Obstetricians and Gynecologists (ACOG) do not recommend a repeat dose of the RSV vaccine in subsequent pregnancies; this consensus is recommended [70, 71]. This position is based on the current lack of sufficient clinical trial data demonstrating the need for a second dose or confirming the duration of protection offered by a single dose in a prior pregnancy. The results of ongoing research, such as the Marguerite Study, may provide new insights and could lead to future changes in these recommendations [72].

### Efficacy and safety of nirsevimab use

The long-acting monoclonal antibody nirsevimab offers consistent, robust, and sustained protection against RSV-related lower respiratory tract infections for at least six months. It reduces hospitalization rates and infant mortality during the first year of life. This benefit provides healthcare systems with a new alternative to reduce RSV-related hospitalizations and mortality in infants under one year of age (quality of evidence: 1A).

### Sufficiency and impact of a single dose of nirsevimab

Clinical trials have demonstrated that a single dose of nirsevimab reduces RSV-related outpatient LRTIs by more than 85% and RSV hospitalizations by 87.5% in healthy infants, both term and preterm, during their first RSV season (quality of evidence: 1A).

### Economic viability of nirsevimab

From a health economics perspective, various cost-effectiveness analyses have concluded that nirsevimab is economically viable in middle-income countries, including Latin America (quality of evidence: 1A).

### Reduction in hospital costs

In Mexico, the economic evaluation model by Comas García et al. projected that its use could significantly reduce hospital costs associated with RSV, especially in contexts with high disease burden and limited intensive care infrastructure [73] (quality of evidence: 1A).

### Coadministration of maternal vaccination and nirsevimab

The standard strategy does not recommend the combined use of maternal vaccination and nirsevimab, except in specific circumstances that justify a potential benefit, such as the following: (1) infants who have lost maternal antibodies, especially those who have undergone procedures such as extracorporeal membrane oxygenation (ECMO); (2) newborns of mothers with chronic immunosuppression, such as transplant recipients or chronic corticosteroid users; (3) infants between eight and 19 months of age with high risk of RSV complications at the start of their second RSV season; (4) children with diseases that increase the risk of severe RSV infection, such as congenital heart diseases, significantly affect hemodynamics.

This recommendation seeks to optimize resources and avoid overimmunization in infants at low or moderate risk (quality of evidence: 1C).

## World Health Organization position and alignment with this consensus

On May 30, 2025, the World Health Organization (WHO), in line with its mandate to provide normative guidance to member states, released its position paper on Immunization to Protect Infants against Respiratory Syncytial Virus Disease [74].

This document, within the time frame established by the consensus methodology and of global relevance, was included in the final review phase of our consensus, allowing for comparison of findings and recommendations. The main points of agreement included the following:

1. The WHO recommends that all countries introduce RSV disease prevention products targeting pregnant women and their infants from birth. Clinical trials, case‒control studies, and real-world data have shown that both the RSVpreF vaccine and nirsevimab are safe, effective, and suitable for use in pregnant women and newborns.
2. For countries choosing maternal vaccination as the primary prevention strategy, the WHO, aligned with our consensus, recommends a single dose of RSVpreF in the third trimester (≥ 28 weeks of gestation), ideally more than two weeks prior to delivery, to ensure sufficient antibody transfer. The WHO also highlights that maternal vaccination may be more programmatically feasible in low-income and middle-income countries and may appear to be more cost-effective than monoclonal antibodies in such nations.
3. Both the WHO and this consensus agree that the only absolute contraindication for RSVpreF or nirsevimab is a history of severe allergic reactions (e.g., anaphylaxis) to any of their components.
4. Similarly, both documents suggest that in high-risk new-borns, a single dose of nirsevimab is not only clinically effective but also cost-effective for reducing the RSV hospitalization burden in infants under six months of age who do not benefit from maternal vaccination.
5. The WHO recommends the use of either maternal vaccination or a long-acting monoclonal antibody, but not for the same mother–infant pair. Exceptions to this recommendation are outlined in both documents [74].
6. The decision to use maternal vaccination or long-acting monoclonal antibodies should consider cost, cost-effectiveness, funding, supply, coverage goals, and feasibility of implementation in each national health system.

## Conclusions

RSV poses a significant burden on infant health in Mexico, contributing substantially to hospitalizations and mortality, particularly in the most vulnerable age groups. The evidence rigorously reviewed by this multidisciplinary expert panel underscores the critical need for effective prevention strategies.

Maternal RSV vaccination with RSVpreF and passive immunization with the long-acting monoclonal antibody nirsevimab have both demonstrated robust efficacy and favorable safety profiles in protecting newborns and infants from severe RSV-related lower respiratory tract infections.

This consensus highlights the importance of timely maternal vaccination during pregnancy, aligning with the current scientific understanding of transplacental antibody transfer kinetics to ensure optimal neonatal protection. Furthermore, it clarifies the appropriate use of nirsevimab, both as a primary preventive measure and in specific circumstances, where maternal antibody transfer may be compromised or in high-risk infants. The experiences of other nations demonstrate the feasibility and significant public health impact of implementing these interventions.

By consolidating the latest epidemiological data, clinical trial outcomes, and real-world effectiveness studies, this document provides actionable, evidence-based recommendations tailored for the Latin American healthcare context. The implementation of these strategies has immense potential for reducing the incidence of severe RSV disease, alleviating the burden on pediatric healthcare services, and ultimately improving infant health outcomes across the nation.

## Data Availability

Data sharing not applicable to this article as no data sets were generated or analysed during the current study.
